# The Effect of Antihypertensive Medication Classes on the Hemodynamic Severity Parameters of Classical and Paradoxical Low-Flow, Low-Gradient Aortic Stenosis

**DOI:** 10.7759/cureus.112455

**Published:** 2026-07-11

**Authors:** Chelsea Grant, Azhar Supariwala

**Affiliations:** 1 Internal Medicine, South Shore University Hospital Northwell Health, Bay Shore, USA; 2 Cardiology, South Shore University Hospital Northwell Health, Bay Shore, USA

**Keywords:** angiotensin-converting enzyme inhibitors, aortic stenosis, classical aortic stenosis, echocardiogram, low flow low gradient, paradoxical aortic stenosis

## Abstract

Background: While the left ventricular (LV) hypertrophy and diffuse interstitial fibrosis characteristic of the pathophysiology of aortic stenosis (AS) are reversed by aortic valve replacement (AVR), the definitive treatment for AS, angiotensin modulators have demonstrated antifibrotic effects in animal models of AS. While goal-directed medical therapy (GDMT) treatment of hypertension is recommended for the early progressive stages of AS, there are no guideline-recommended antihypertensive therapies for stage D low-flow, low-gradient (LFLG) severe AS.

Methods: All echocardiographic studies performed at South Shore University Hospital between January 2020 and December 2024 were retrospectively reviewed to identify a cohort of patients with LFLG severe AS. The progression of echocardiographic hemodynamic severity parameters was then examined according to prescribed antihypertensive medications in this cohort of 218 patients followed for a mean period of 19.1 months.

Results: In this cohort, the prevalence of LFLG severe AS with preserved left ventricular ejection fraction (LVEF) was 72.9%. Angiotensin-converting enzyme (ACE) inhibitors were not associated with any statistically significant change in hemodynamic severity markers, whereas angiotensin receptor blockers (ARBs) were associated with a statistically significant increase in peak jet velocity in a subset of the cohort (p = 0.042).

Conclusion: In this cohort, angiotensin modulators were not associated with slower progression of AS severity parameters. Rather, the GDMT members - ​​​​sodium-glucose cotransporter 2 (SGLT2) inhibitors and beta-blockers - were associated with differential reductions in stenosis severity markers in patients with LFLG severe AS.

## Introduction

According to the 2020 American College of Cardiology (ACC)/American Heart Association (AHA) guidelines, there are four stages of aortic stenosis (AS) progression, ranging from risk for AS at stage A, to progressive mild-to-moderate AS at stage B, to asymptomatic severe AS at stage C, and to symptomatic severe AS at stage D [1]. Stage D is further subdivided into three classes based on transvalvular gradient and left ventricular ejection fraction (LVEF), with D1 representing high-gradient AS, D2 representing low-flow, low-gradient (LFLG) AS with reduced LVEF, and D3 representing LFLG AS with preserved LVEF. Hemodynamic severity is best depicted by peak transaortic velocity (Vmax) calculated by the Bernoulli equation and the mean transvalvular pressure gradient (MPG) calculated by the continuity equation during Doppler echocardiography or cardiac catheterization. Progression leading to eventual symptom onset occurs in asymptomatic AS (Classes A, B, and C) once the Vmax reaches 2 m/s, with an annual increase in Vmax of approximately 0.3 m/s, an increase in mean pressure gradient of 7-8 mmHg, and an annual decrease in aortic valve area (AVA) of 0.15 cm². In contrast to D1, which is characterized by a Vmax greater than or equal to 4 m/s, an MPG greater than or equal to 40 mmHg, and an AVA less than or equal to 1 cm², stages D2 and D3 demonstrate a Vmax less than 4 m/s and a mean gradient less than 40 mmHg. The low flow in stages D2 and D3 is further defined by a stroke volume (SV) indexed to body surface area (BSA) of less than 35 mL/m², which anatomically correlates with left ventricular (LV) hypertrophy, reduced LV chamber size, and diastolic dysfunction. Another way of characterizing the LFLG stages of AS is by an AVA indexed to BSA of less than or equal to 0.6 cm²/m².

Both stage D2 (classical LFLG AS) and stage D3 (paradoxical LFLG AS) are characterized by higher LV hemodynamic load, greater valvuloarterial impedance, lower transvalvular flow, concentric ventricular hypertrophy, decreased SV, decreased cardiac output, and decreased LV fractional shortening consistent with intrinsic myocardial dysfunction [2]. Higher LV loading due to atherosclerotic rigid arterial walls with decreased compliance culminates in systolic hypertension and increased afterload, to which the LV responds by increasing wall thickness to mitigate wall stress according to Laplace's law. In other words, fibrocalcific remodeling of the aortic valve leaflets results in tandem narrowing of the valve annulus and increased afterload with an LV hypertrophic response. When this compensatory mechanism becomes overwhelmed over the course of years or even decades, additional hypertrophy of the LV transitions to heart failure and symptomatic cardiovascular events. Progression from aortic valve sclerosis to valve obstruction occurs in 10%-15% of patients over a two- to five-year timeframe, and symptom onset is associated with an average survival of two to three years without valve replacement [3].

Quantification of the aortic valve calcium score by coronary CT imaging is applied in the prognostication of D2 and D3 AS, with Agatston scores of 1300 in women and 2000 in men qualifying as severe AS [1]. Low-density lipoprotein (LDL) accumulates in the subendothelial layer of the aortic valve, becomes oxidized, and triggers a chronic inflammatory response that recruits macrophages, T cells, and mast cells to the area [4]. Incited by this inflammatory process, valve interstitial fibroblast-like cells harbored in the aortic valve produce disorganized collagen lattices, causing valve fibrosis and driving a cycle of calcification similar to bone formation. Statin therapy is therefore indicated for primary and secondary prevention of atherosclerosis in all patients with calcific AS but is not indicated for preventing the hemodynamic progression of AS, as demonstrated by randomized controlled trials [4]. Interestingly, angiotensin-converting enzyme (ACE) is trafficked by LDL and generates angiotensin II, which contributes to the inflammatory fibrotic milieu.

The LV hypertrophy and diffuse interstitial fibrosis characteristic of AS pathophysiology are reversed by aortic valve replacement (AVR), as long as mid-wall fibrosis of tissue has not occurred. Surgical aortic valve replacement (SAVR) is recommended for adults younger than 65 years with prolonged life expectancy and for those without significant comorbidities, whereas treatment options broaden to include transcatheter aortic valve replacement (TAVR) in adults older than 65 years [1]. Valve replacement is indicated not only in stage D AS but also in asymptomatic severe AS (stage C1) with serum B-type natriuretic peptide levels three times the limit of normal, in asymptomatic severe AS with an LVEF less than 50% (stage C2), in patients with an abnormal exercise-induced blood pressure drop of at least 10 mmHg, in those with rapid hemodynamic progression defined by an increase in aortic velocity greater than 0.3 m/s per year, or in those with a Vmax greater than 5 m/s. Percutaneous balloon aortic valvuloplasty (BAV) plays a bridging role to aortic valve intervention by fracturing leaflet calcific deposits and expanding the structural annulus. This means of dilating the aortic valve, however, rarely expands AVA beyond 1 cm2, results in a moderate reduction in transvalvular pressure gradient, and leads to severe aortic regurgitation (AR) with clinical deterioration within 6 to 12 months in most patients.

It is recommended that blood pressure control be optimized before hemodynamic assessment of D2 and D3 AS because systemic hypertension imparts a pressure load on the LV, obstructs the aortic valve, and lowers both forward SV and the transvalvular pressure gradient [1]. While there has been concern that antihypertensives such as diuretics may result in decreased cardiac output and SV in AS, no studies recommend specific therapies for the management of hypertension in AS. However, standard goal-directed medical therapy (GDMT) is recommended for hypertension treatment in AS stages A, B, and C. In terms of stage D, it turns out that renin-angiotensin-aldosterone inhibitors are associated with lower one-year mortality following TAVR among patients with preserved LVEF (D3) AS but not among those with stage D2 AS and reduced LVEF. Without formal consensus on the treatment strategies before valve replacement for stages D2 and D3 AS, this study is undertaken to explore the effects of various antihypertensive agents on AS hemodynamics.

## Materials and methods

The study protocol followed the principles outlined in the Declaration of Helsinki for human studies and was approved by the Institutional Review Board of the Feinstein Institute of North Shore University Hospital. A waiver of informed consent for this retrospective observational study was granted with protocol approval.

Patients 

All echocardiographic studies performed at South Shore University Hospital are retrospectively reviewed to identify a cohort of patients with severe AS defined as an AVA ≤ 1 cm^2^. Of 9,743 patients with at least two echocardiogram studies, 1,186 patient echocardiograms were identified with the diagnosis of LFLG severe AS. Incomplete echocardiograms, patients aged ≤ 18 years, those with prosthetic valves, and those with complex congenital heart disease were excluded. Additional exclusion criteria during this screening process included moderate or severe concomitant valvulopathy (aortic, mitral, or tricuspid regurgitation/stenosis), an inadequate interval between serial echocardiograms (<6 months), and an echocardiogram performed outside the study year range (January 2020 to December 2024). Patients were then divided into two subsets: those with low-gradient (LG; <40 mmHg) severe AS (AVA ≤ 1 cm²) with preserved EF (>50%), defined as paradoxical LFLG AS, and those with LG severe AS with reduced EF (<50%), defined as classical LFLG AS. Medical records were reviewed for comorbidities, including hyperlipidemia (HLD), atrial fibrillation, coronary artery disease (CAD), diabetes mellitus (DM), cerebrovascular disease (CVD), chronic kidney disease (CKD), and chronic obstructive pulmonary disease (COPD). Clinical data, including age, sex, and blood pressure, were also extracted. Aortic valve intervention, BAV, TAVR, or SAVR was determined by reviewing the medical records.

Echocardiography 

All patients underwent comprehensive Doppler echocardiographic studies using commercially available ultrasound systems. Experienced sonographers performed all examinations. AVA was determined using the continuity equation. Peak and mean transvalvular pressure gradients were derived using the modified Bernoulli equation. Peak jet velocity was measured using the continuous-wave Doppler technique. Ejection fraction was determined from the apical four-chamber and apical two-chamber views using the biplane Simpson’s formula. Preserved EF was defined as EF ≥ 50% according to the 2022 AHA/ACC/Heart Failure Society of America (HFSA) Guideline for the Management of Heart Failure [5].

Statistical analysis

Continuous variables are presented as means with standard deviations. Categorical variables are presented as absolute numbers and percentages. Baseline demographic and echocardiographic variables were compared between the paradoxical and classical LFLG groups using Student's t-test and chi-square test, as appropriate. Analyses were stratified according to antihypertensive therapy using linear mixed models fitted with medication class as the nominal factor and follow-up time as the continuous covariate. A linear mixed model of repeated measurements with a random-effects intercept was used to evaluate changes in the echocardiographic parameters over time while controlling for heterogeneous follow-up durations of all patients. Statistical significance was defined as p < 0.05. All analyses and plots were performed using Jamovi version 2.7.24 (The Jamovi Project, Sydney, Australia).

## Results

Baseline clinical, demographic, and echocardiographic characteristics

**Figure 1:**
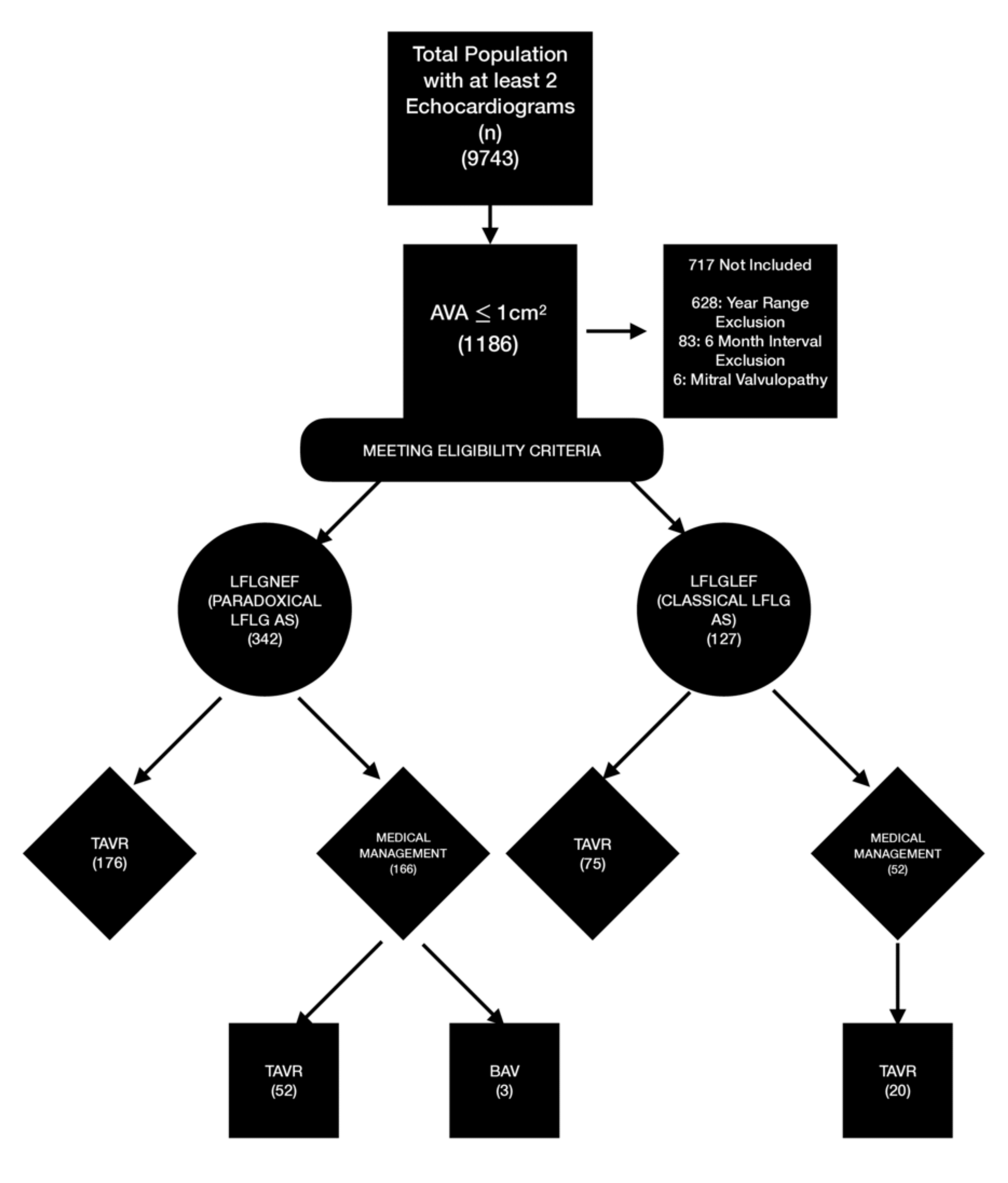
Schematic flow chart of the study AVA: Aortic valve area; LFLGNEF: Low-flow, low-gradient with normal ejection fraction; LFLGLEF: Low-flow, low-gradient with low ejection fraction; TAVR: Transcutaneous aortic valve repair; BAV: Balloon aortic valvuloplasty.

Of the 1,186 patients with an AVA ≤ 1 cm², 176 patients were classified with paradoxical LFLG AS and 75 patients with classical LFLG AS underwent TAVR based on baseline hemodynamic parameters (Figure 1).

The study population therefore consisted of 218 patients, including 101 (46.3%) men and 117 (53.7%) women. The mean age was 78.8 years, with no significant difference between the classical and paradoxical AS subgroups (Table 1). A statistically significant greater proportion of patients with stage D2 classical LFLG AS had CKD, CAD, and HLD. There were no statistically significant differences in the prescription of ACE inhibitors (ACEi), angiotensin receptor blockers (ARBs), sodium-glucose cotransporter 2 (SGLT2) inhibitors, diuretics, or vasodilators between the two groups. Blood pressure (p = 0.017), peak jet velocity (p = 0.046), and mean pressure gradient (p = 0.036) were significantly higher in patients with paradoxical LFLG AS at the time of investigation. Per diagnostic definition, LVEF was significantly lower in patients with classical LFLG AS (p < 0.001). 

**Table 1:** Baseline characteristics for patient groups This table shows the baseline characteristics of the D2 and D3 low-flow, low-gradient AS groups. Values are reported as mean ± SD or n (%). LFLGNEF: Low-flow, low-gradient normal ejection fraction; LFLGLEF: Low-flow, low-gradient low ejection fraction; CKD: Chronic kidney disease; CVA: Cerebrovascular accident; CAD: Coronary artery disease; HLD: Hyperlipidemia; COPD: Chronic obstructive pulmonary disease; CCB: Calcium channel blocker; BB: Beta blocker; ACEi: Angiotensin-converting enzyme inhibitor; ARB: Angiotensin receptor blocker; SGLT2: Sodium glucose cotransporter 2 blockers; ARNi: Angiotensin neprilysin inhibitor.

Variable	LFLGNEF (Paradoxical LFLG AS) (N = 166)	LFLGLEF (Classical LFLG AS) (N = 52)	p-value
Length of follow-up (months)	19.3 ± 13.7	18.9 ± 13.3	0.849
Age (Years)	80 ± 9.1	77.6 ± 10.4	0.113
Male	69 (41.5%)	32 (61.5%)	0.012
Systolic BP (mmHg)	140.6 ± 24.8	131.4 ± 22.1	0.017
Comorbidities			
Diabetes	60 (36.1%)	20 (38.5%)	0.762
CKD	40 (24%)	22 (42.3%)	0.011
Dialysis	11 (6.6%)	9 (17.3%)	0.020
Atrial fibrillation	69 (41.6%)	24 (46.2%)	0.559
CVA	21 (12.7%)	6 (11.5%)	0.921
CAD	77 (46.4%)	40 (76.9%)	<0.001
HLD	138 (83.1%)	50 (96.2%)	0.049
COPD	23 (13.9%)	10 (19.2%)	0.345
BP medications			
CCB	68 (41%)	7 (13.5%)	<0.001
BB	105 (63.3%)	43 (82.7%)	0.009
ACEi	26 (15.7%)	3 (5.8%)	0.067
ARB	39 (23.5%)	10 (19.2%)	0.520
SGLT2	14 (8.4%)	9 (17.3%)	0.069
ARNi	3 (1.8%)	11 (21.2%)	<0.001
Diuretic	86 (51.8%)	34 (65.4%)	0.086
Vasodilator	25 (15.1%)	10 (19.2%)	0.475
Echocardiographic data			
Aortic valve area (cm^2^)	0.86 ± 0.16	0.85 ± 0.18	0.532
Peak jet velocity (m/s)	3.1 ± 0.78	2.87 ± 0.61	0.046
Mean gradient (mmHg)	22.6 ± 12.5	18.7 ± 7.8	0.036
Ejection fraction (%)	63.7 ± 11.4	34.8 ± 9.7	<0.001

Progression of AS severity parameters

**Figure 2:**
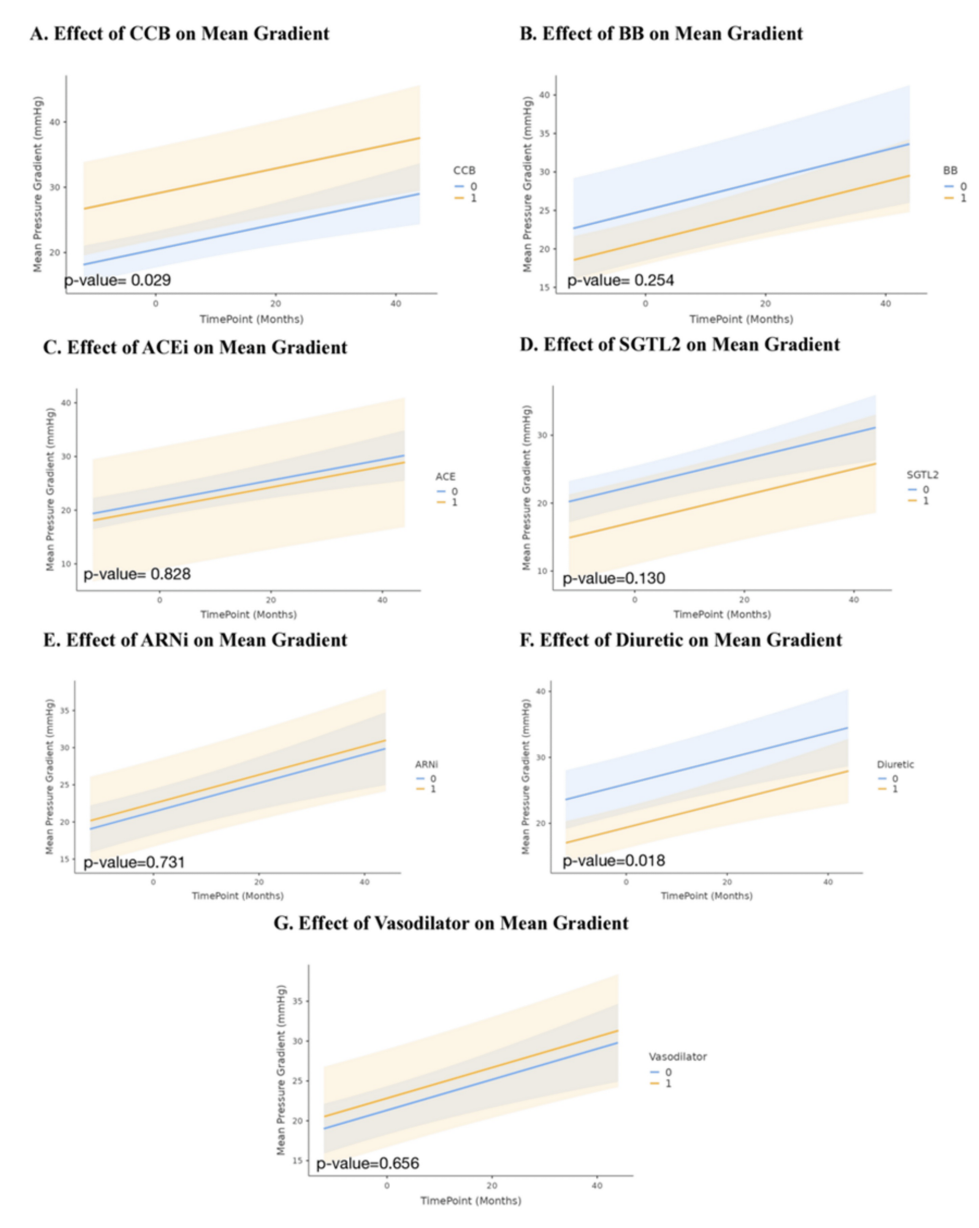
Effect of various medication classes on aortic stenosis mean pressure gradient in classical LFLG AS 0 = medication class not in use; 1 = medication class in use. (A) Effect of CCB, (B) effect of BB, (C) effect of ACEi, (D) effect of SGLT2 inhibitors, (E) effect of ARNi, (F) effect of diuretics, and (G) effect of vasodilators. LFLG: Low-flow, low-gradient; AS: Aortic stenosis; SGLT2: Sodium-glucose cotransporter 2 blockers; CCB: Calcium channel blocker; BB: Beta blocker; ACEi: Angiotensin-converting enzyme inhibitor; ARNi: Angiotensin receptor-neprilysin inhibitor.

**Figure 3:**
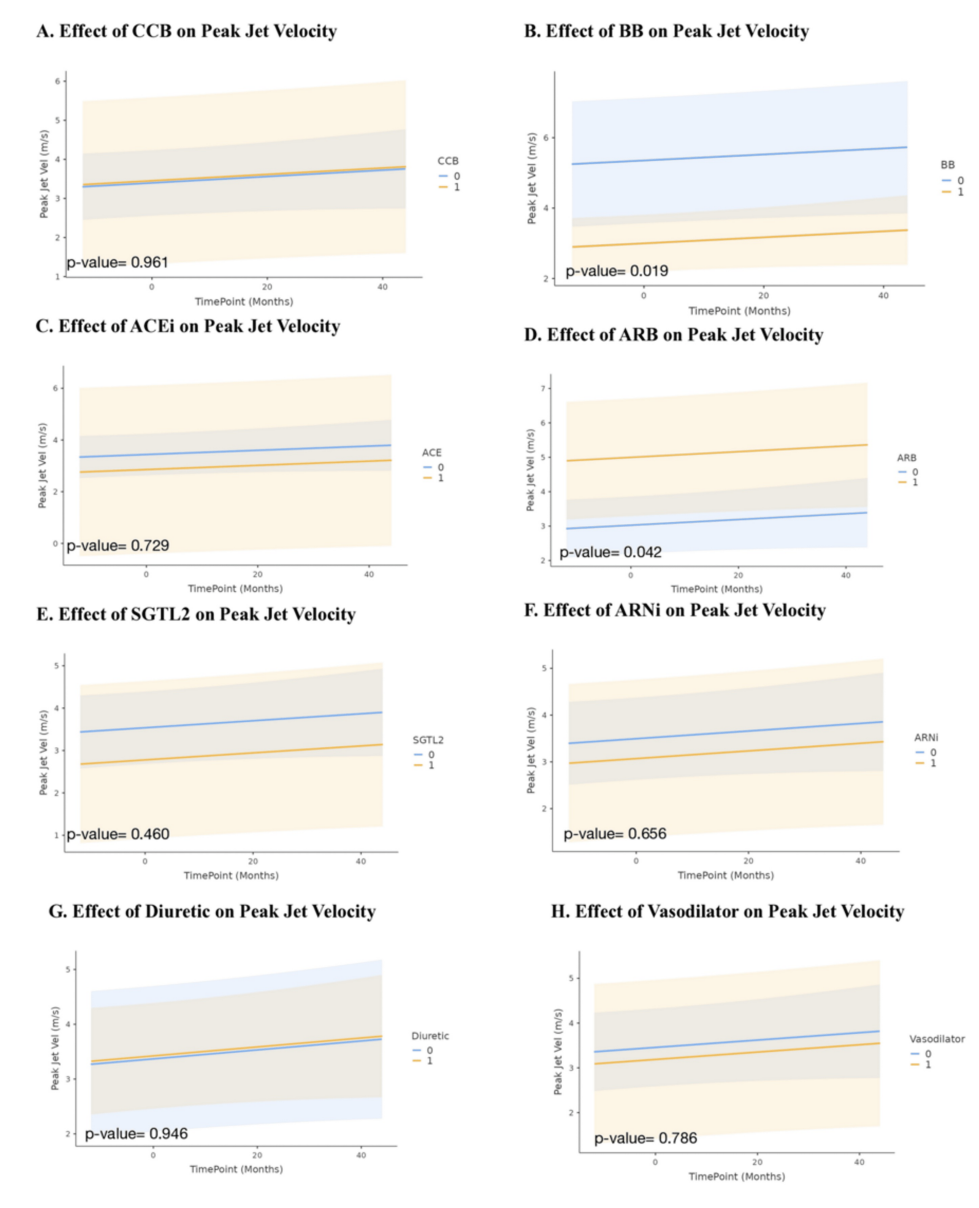
Effect of various medication classes on aortic stenosis peak jet velocity in classical LFLG AS 0 = medication class not in use; 1 = medication class in use. (A) Effect of CCB, (B) effect of BB, (C) effect of ACEi, (D) effect of ARB, (E) effect of SGLT2, (F) effect of ARNi, (G) effect of diuretic, (H) effect of vasodilator. LFLG: Low-flow, low-gradient; AS: Aortic stenosis; SGLT2: Sodium-glucose cotransporter 2 blockers; CCB: Calcium channel blocker; BB: Beta blocker; ACEi: Angiotensin-converting enzyme inhibitor; ARNi: Angiotensin receptor-neprilysin inhibitor; ARB: Angiotensin receptor blocker.

**Figure 4:**
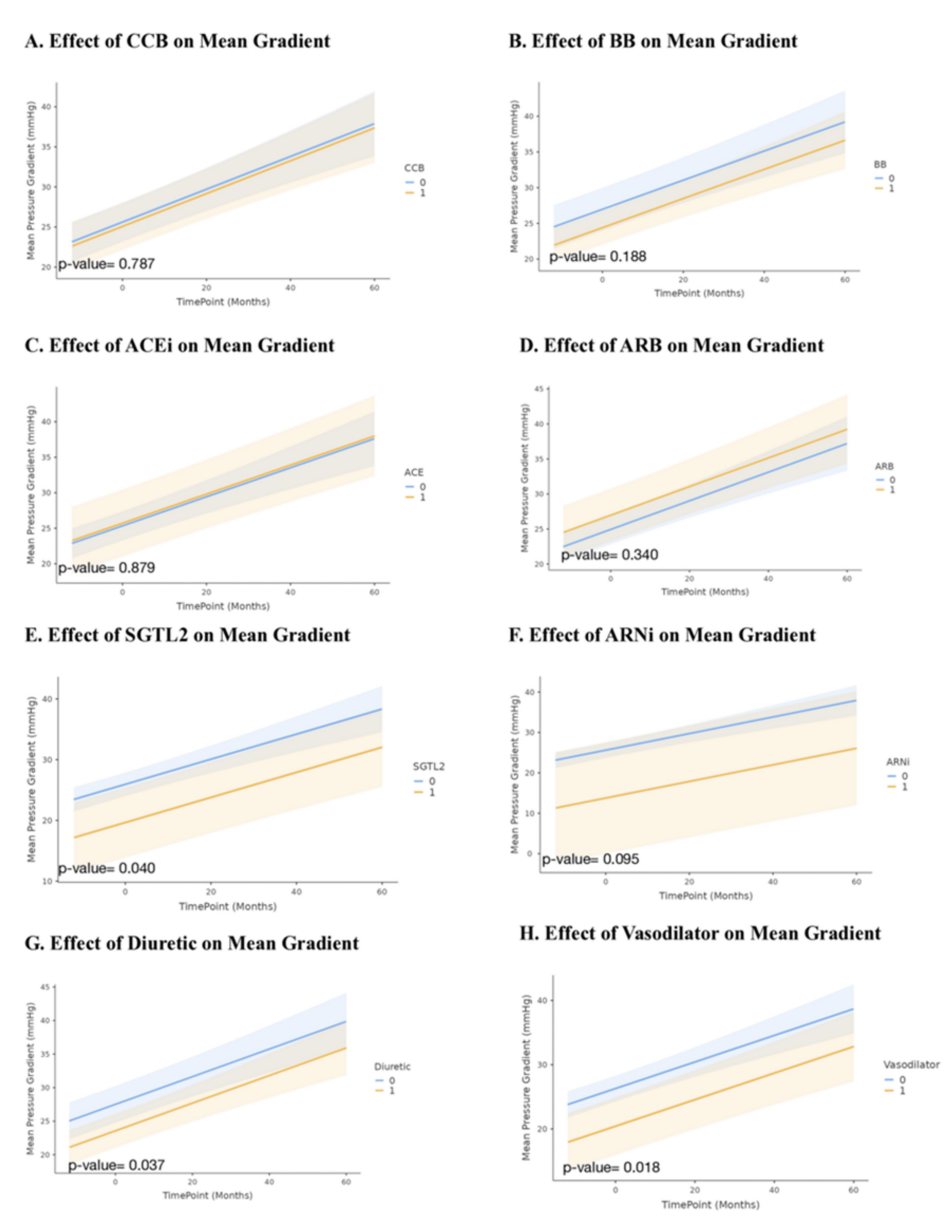
Effect of various medication classes on aortic stenosis mean pressure gradient in paradoxical LFLG AS 0 = medication class not in use; 1 = medication class in use. (A) Effect of CCB, (B) effect of BB, (C) effect of ACEi, (D) effect of ARB, (E) effect of SGLT2 inhibitors, (F) effect of ARNi, (G) effect of diuretics, (H) effect of vasodilators. LFLG: Low-flow, low-gradient; AS: Aortic stenosis; SGLT2: Sodium-glucose cotransporter 2 blockers; CCB: Calcium channel blocker; BB: Beta blocker; ACEi: Angiotensin-converting enzyme inhibitor; ARNi: Angiotensin receptor-neprilysin inhibitor; ARB: Angiotensin receptor blocker.

**Figure 5:**
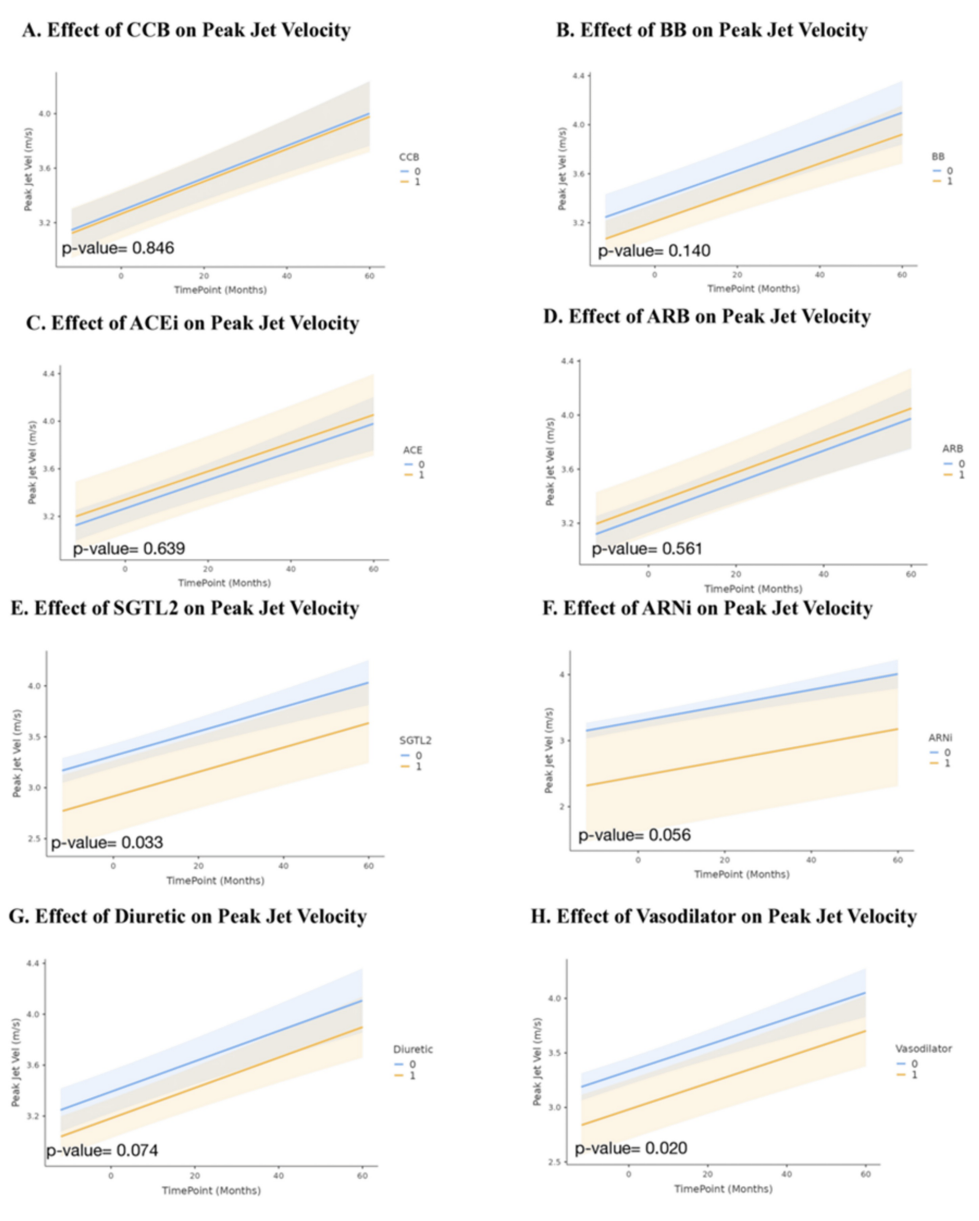
Effect of various medication classes on aortic stenosis peak jet velocity in paradoxical LFLG AS 0 = medication class not in use; 1 = medication class in use. (A) Effect of CCB, (B) effect of BB, (C) effect of ACEi, (D) effect of ARB, (E) effect of SGLT2 inhibitors, (F) effect of ARNi, (G) effect of diuretics, (H) effect of vasodilators. LFLG: Low-flow, low-gradient; AS: Aortic stenosis; SGLT2: Sodium-glucose cotransporter 2 blockers; CCB: Calcium channel blocker; BB: Beta blocker; ACEi: Angiotensin-converting enzyme inhibitor; ARNi: Angiotensin receptor-neprilysin inhibitor; ARB: Angiotensin receptor blocker.

The mean follow-up period was 19.1 months, with no significant difference between the groups. Figures 2-5 graphically represent the average changes over time in AS peak jet velocity and mean gradient severity parameters according to the baseline classifications of stage D2 classical and stage D3 paradoxical LFLG AS. 

**Figure 6:**
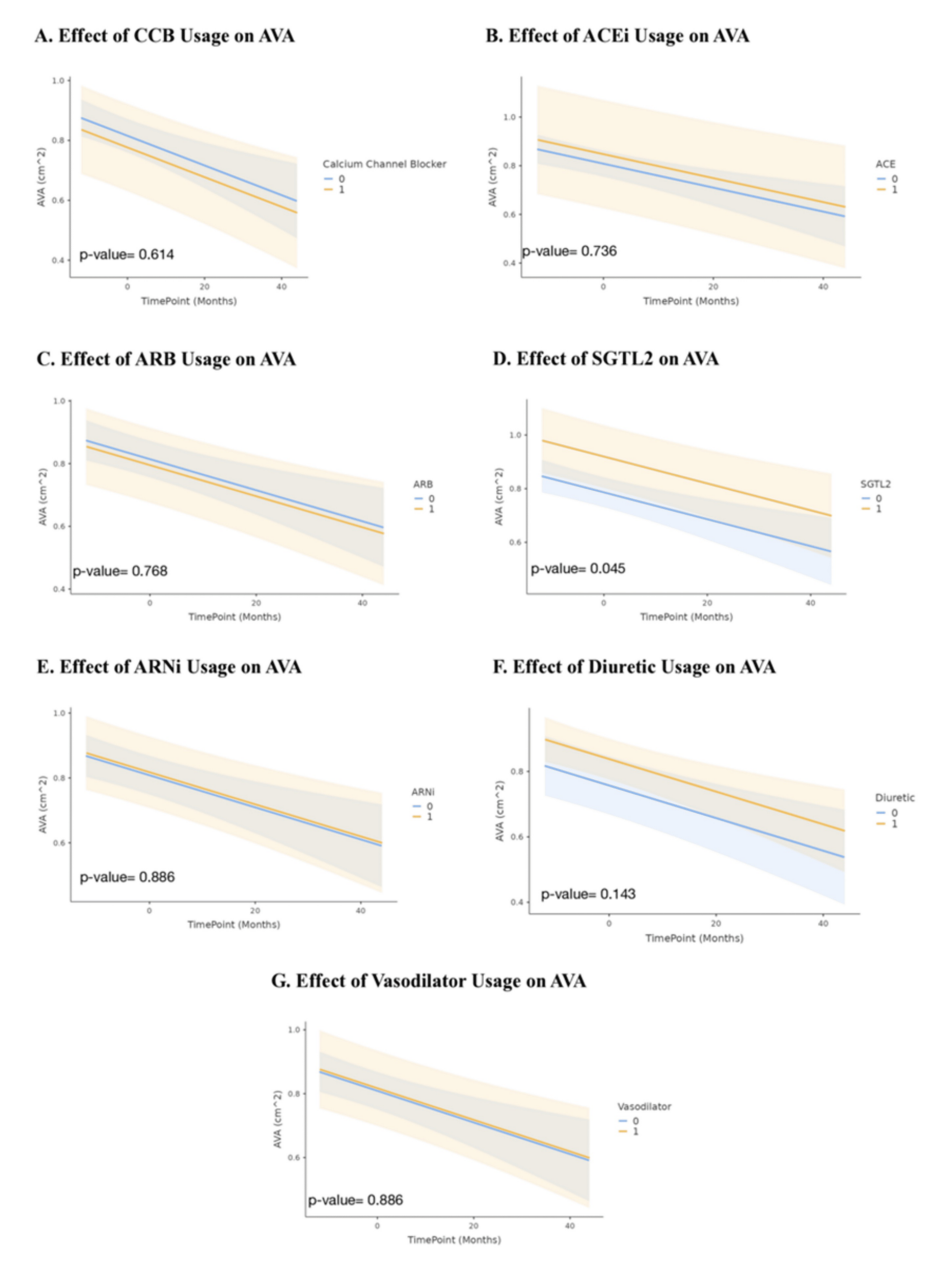
Effect of various medication classes on aortic stenosis aortic valve area in classical LFLG AS 0 = medication class not in use; 1 = medication class in use. (A) Effect of CCB, (B) effect of ACE inhibitors, (C) effect of ARB, (D) effect of SGLT2 inhibitors, (E) effect of ARNi, (F) effect of diuretics, and (G) effect of vasodilators. LFLG: Low-flow, low-gradient; AS: Aortic stenosis; SGLT2: Sodium-glucose cotransporter 2 blockers; CCB: Calcium channel blocker; BB: Beta blocker; ACEi: Angiotensin-converting enzyme inhibitor; ARNi: Angiotensin receptor-neprilysin inhibitor; ARB: Angiotensin receptor blocker.

**Figure 7:**
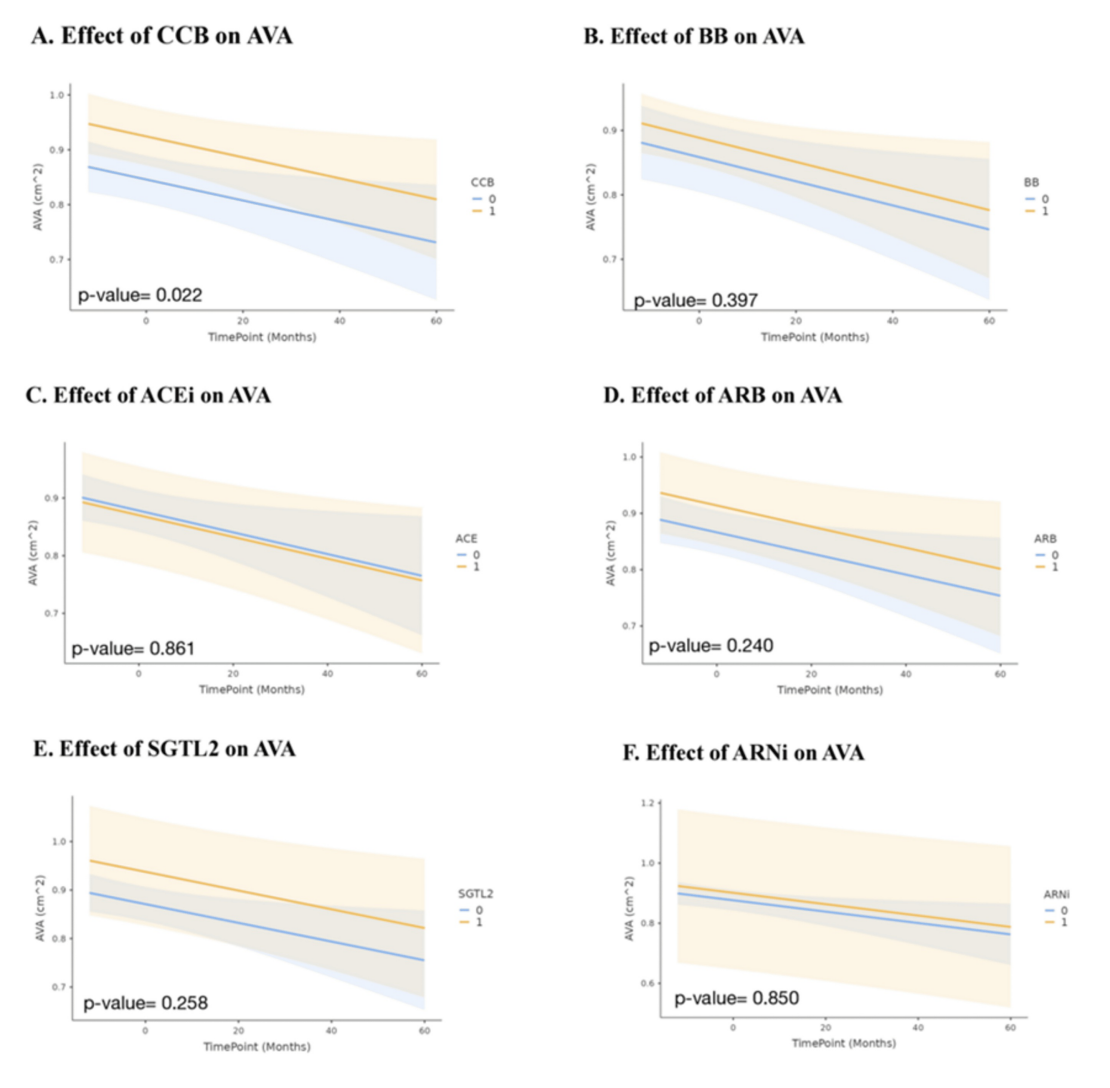
Effect of various medication classes on aortic stenosis aortic valve area in paradoxical LFLG AS 0 = medication class not in use; 1 = medication class in use. (A) Effect of CCB, (B) effect of ACE inhibitors, (C) effect of ARB, (D) effect of SGLT2 inhibitors, (E) effect of ARNi, (F) effect of diuretics, and (G) effect of vasodilators. LFLG: Low-flow, low-gradient; AS: Aortic stenosis; SGLT2: Sodium-glucose cotransporter 2 blockers; CCB: Calcium channel blocker; BB: Beta blocker; ACEi: Angiotensin-converting enzyme inhibitor; ARNi: Angiotensin receptor-neprilysin inhibitor; ARB: Angiotensin receptor blocker.

Graphical representations of AS AVA progression stratified by medication class are shown in Figures 6, 7.

Table 2 depicts the comparison of these changes stratified by medication class for both the D2 and D3 groups. 

**Table 2:** Effect of medication classes on aortic stenosis severity parameters over time This table shows the changes over time in aortic stenosis severity parameters by medication group, as depicted using linear mixed models with the respective coefficients. LFLG: Low-flow, low-gradient aortic stenosis; AVA: Aortic valve area (cm²); MPG: Mean pressure gradient (mmHg); peak jet vel: Peak jet velocity (m/s); CCB: Calcium channel blocker; BB: Beta-blocker; ACEi: Angiotensin-converting enzyme inhibitor; ARB: Angiotensin receptor blocker; SGLT2: Sodium-glucose cotransporter 2 blocker; ARNi: Angiotensin receptor-neprilysin inhibitor.

	Paradoxical LFLG AS coefficients and P-values						Classical LFLG AS coefficients and P-values					
Med class	AVA (cm^2^)	P-value	MPG (mmHg)	P-value	Peak jet vel (m/s)	P-value	AVA (cm^2^)	P-value	MPG (mmHg)	P-value	Peak jet vel (m/s)	P-value
CCB	0.078	0.022	-0.506	0.787	-0.023	0.846	-0.039	0.614	3.79	0.029	0.056	0.961
BB	0.031	0.397	-2.56	0.188	-0.177	0.140	-6.48e-4	0.993	-4.097	0.254	-2.35	0.0194
ACEi	-0.009	0.861	0.402	0.879	0.075	0.639	0.039	0.736	-1.273	0.828	-0.581	0.728
ARB	0.044	0.240	2.66	0.340	0.119	0.561	-0.019	0.768	0.039	0.991	1.972	0.042
SGLT2	0.061	0.258	-6.61	0.040	-0.415	0.033	0.132	0.045	-5.308	0.130	-0.757	0.460
ARNi	0.024	0.850	-11.83	0.095	-0.832	0.056	0.009	0.886	1.127	0.731	-0.424	0.656
Diuretic	0.047	0.173	-3.94	0.037	-0.208	0.074	0.080	0.143	-6.55	0.018	0.055	0.946
Vasodilator	0.021	0.659	-6.005	0.018	-0.355	0.020	0.007	0.886	1.525	0.656	-0.267	0.786

Dihydropyridine calcium channel blockers, such as amlodipine, were associated with a statistically significant increase in AVA in D3 paradoxical AS (p = 0.022) and a detrimental increase in MPG in classical AS (p = 0.029). Beta-blockers were associated with a statistically significant decrease in peak jet velocity in D2 classical AS (p = 0.019). ACEi were not associated with any statistically significant changes in hemodynamic severity markers in either subgroup, whereas ARBs were associated with a statistically significant detrimental increase in peak jet velocity in patients with classical LFLG AS (p = 0.042). SGLT2 inhibitors, such as Jardiance and Farxiga, were associated with statistically significant decreases in mean pressure gradient and peak jet velocity in D3 AS (p = 0.040 and 0.033, respectively), as well as an increase in AVA in D2 AS (p = 0.045). Diuretics were associated with statistically significant decreases in mean pressure gradients for both paradoxical and classical LFLG AS, whereas vasodilators, such as hydralazine and isosorbide mononitrate, were associated with decreases in both mean pressure gradient and peak jet velocity in D3 LFLG AS.

Of the 166 medically managed patients with paradoxical LFLG AS, 52 (31.3%) subsequently underwent TAVR and three (1.8%) underwent BAV. Of the 52 medically managed patients with classical LFLG AS, 20 (38.5%) eventually underwent TAVR. The decision to proceed with procedural intervention was left to the referral cardiologist.

## Discussion

Low-flow, low-gradient paradoxical AS is paradoxical in that there is preserved EF with decreased SV. This variant of severe AS occurs in 5%-25% of patients with stage D AS [3]. The SEAS trial, a randomized controlled trial comparing the effects of combined simvastatin-ezetimibe treatment versus placebo in 1,873 patients, found a prevalence of 29% for LFLG AS in the study population [6]. Another longitudinal multicenter observational study in Spain, following 903 patients over a median follow-up period of 60.2 months, found a prevalence of 10.7% for paradoxical LFLG severe AS, with progression to AVR in 51.5% of this subset [7]. At baseline, the mean AVA was 0.86 ± 0.16 cm², the peak aortic velocity was 3.1 ± 0.78 m/s, and the mean aortic gradient was 22.6 ± 12.5 mmHg in this cohort. In another retrospective echocardiographic study of 512 cases of severe AS with an indexed AVA ≤ 0.6 cm²/m² at the Quebec Heart Institute, the prevalence of paradoxical LFLG AS was 35% [8]. In this LFLG population with preserved LVEF, the mean AVA was 0.76 ± 0.23 cm², the peak aortic velocity was 3.5 ± 0.9 m/s, the MPG was 32 ± 17 mmHg, and the mean EF was 62% ± 8%. In a prospective study of 349 patients with severe AS, Maes et al. reported a significant increase in the mean aortic gradient in 205 patients with paradoxical LFLG AS during a median follow-up period of 28 months [9]. Of the 102 paradoxical LFLG AS patients with follow-up echocardiographic data, the mean gradient increased from 29 ± 6 to 38 ± 11 mmHg, with 45% of this subset progressing to high-gradient D1 AS. Tribouilloy et al. further demonstrated that LFLG AS progressed to high-gradient stage D1 AS in 40% of patients in a prospective study following 57 patients over a median follow-up period of 39 months [10]. In the present study, paradoxical LFLG AS accounted for 72.9% of patients with LFLG AS.

In animal models, aldosterone, along with the other two arms of the renin-angiotensin-aldosterone system (RAAS), promotes the perivascular inflammation and interstitial myocardial fibrosis characteristic of AS pathophysiology. Reversal of LV hypertrophy has been observed with the use of RAAS receptor antagonists in these animal models [11]. In a randomized, double-blind study conducted in 65 patients with paradoxical AS, Stewart et al. demonstrated that eplerenone was not correlated with slowed progression of AS [12]. In another randomized controlled trial studying the effect of candesartan in 51 patients with severe AS awaiting AVR, this ARB had no significant effect on exercise tolerance or echocardiographic LV structure or function after a short five-month follow-up period [13]. Limitations of this study include the short follow-up period and the late stage of disease examined. A study examining the extent of tissue remodeling in stenotic aortic valves explanted at the time of AVR revealed significantly lower valve weight and remodeling scores in patients taking ARBs [14]. In a retrospective observational study that divided 338 patients with moderate-to-severe AS into categories based on blood pressure and use of ACEi versus ARBs, ARBs, but not ACEi, were significantly correlated with both decreased mortality risk and slower progression of annualized peak jet velocity [15]. A larger retrospective study in Scotland following 2,117 patients with and without ACEi or ARB prescriptions found that both medication classes were correlated with significantly lower all-cause mortality (hazard ratio, 0.76; p < 0.0001) and cardiovascular events (hazard ratio, 0.77; p < 0.0001) [16]. Enalapril, in particular, demonstrated symptomatic improvement in six-minute walk distance in a randomized, double-blind, controlled trial enrolling 56 patients with AS [17].

Second-generation dihydropyridine calcium channel blockers are considered safe in AS because they do not depress LV function [18]. Diuretics, however, are recommended to be started at low doses because of their capacity to reduce LV diastolic filling. Beta-blockers are recommended in AS with concomitant CAD, and metoprolol succinate demonstrated statistically significant reductions of 7 mmHg (p = 0.05) and 4 mmHg (p = 0.03) in peak and mean aortic valve gradients, respectively, in a double-blind study of 40 patients followed for 22 weeks [19,20]. The present study did not find statistically significant reductions in AS severity parameters associated with ACEi or ARB use. SGLT2 inhibitors, however, were correlated with statistically significant reductions in peak jet velocity and mean pressure gradient in paradoxical LFLG AS. In addition, diuretics and vasodilators were correlated with reduced mean pressure gradients in both D2 and D3 classifications of severe AS.

Limitations

The present study was retrospective, and the data did not allow identification of the precise timing of symptom onset during the disease course or the incidence of patient comorbidities. The retrospective nature of the study also introduced various confounding factors, including differential frequency of patient follow-up, unstandardized measures of individual medication compliance, and unstandardized echocardiographic reporting cultures. Current recommendations support a multipronged approach for diagnosing LFLG that confirms severity by SV indexed to BSA, in addition to aortic calcium score measured by CT, neither of which was systematically evaluated in the present study. Data regarding the indication, dose, onset, and duration of the various medication therapies were not included in the study protocol, limiting the ability to discriminate dose and duration effects via statistical analysis. Statistical analysis was further limited by the lack of adjustment for these factors, as well as for the medical comorbidities of each individual, for patient demographics, BSA-indexed SV, AVA indexed to BSA, and LVEF.

## Conclusions

Indicated as GDMT for heart failure with preserved EF, SGLT2 inhibitors are correlated with slowed progression of AS severity parameters in a subset of patients with severe AS and preserved EF. Symptomatic adjuncts to GDMT, including diuretics and vasodilators, are correlated with lower mean pressure gradients in both D2 and D3 LFLG AS. Angiotensin modulators, though historically linked to reduction of LV fibrosis pathways, do not demonstrate a correlation with reduced progression of AS hemodynamic severity parameters in this cohort over a mean follow-up period of 19.1 months.
